# New species of the *Rhaconotusjacobsoni* group (Hymenoptera, Braconidae, Doryctinae) from Vietnam

**DOI:** 10.3897/zookeys.853.33938

**Published:** 2019-06-06

**Authors:** Nguyen Thi Oanh, Khuat Dang Long

**Affiliations:** 1 Dong Thap University, Cao Lanh city, Dong Thap, Vietnam Dong Thap University Dong Thap Vietnam; 2 Institute of Ecology and Biological Resources (IEBR), Vietnam Academy of Science and Technology (VAST), 18 Hoang Quoc Viet Road, Ha Noi, Vietnam Vietnam Academy of Science and Technology Ha Noi Vietnam

**Keywords:** Ichneumonoidea, Rhaconotini, Afrotropical, Oriental, systematics

## Abstract

Four new species of the genus *Rhaconotus* Ruthe from Vietnam are described and illustrated – *Rhaconotusdirectus* Long, **sp. nov**., *R.laevigatus* Long, **sp. nov.**, *R.robustus* Long, **sp. nov.**, and *R.simulatus* Long, **sp. nov.** A key to species of *Rhaconotusjacobsoni* group from the Oriental region is provided.

## Introduction

*Rhaconotus* Ruthe, 1854 is one of the largest genera of the doryctine tribe Rhaconotini, with more than 100 described species (Belokobylskij 2001; Jasso-Martínez et al. 2019). Most of the known species of *Rhaconotus* occur in the Oriental and Afrotropical regions (Belokobylskij and Chen 2004; Belokobylskij and Maetô 2009; Yu et al. 2012), and recently Belokobylskij and Zaldívar-Riverón (2015) described four new species of this genus from Neotropical region.

*Rhaconotusjacobsoni* group is one of several groups of this genus divided by Belokobylskij (2001) and separated from other *Rhaconotus* groups by having metasoma with six visible tergites and length of first metasomal tergite 2.3–2.8 × apical width. Currently, this group contains four described species from the Oriental region; of those only one species, *R.thayi* Belokobylskij, was known from Vietnam (Yu et al. 2013; Belokobylskij and Chen 2004; Long and Belokobylskij 2003). In this paper, four new species of the *Rhaconotusjacobsoni* group from Vietnam are described.

## Materials and methods

The specimens were mainly collected in Malaise traps and some by using sweep nets. The material was stored in 70% or 96% ethanol, prepared with the AXA method (van Achterberg 2009; van Achterberg et al. 2010) and glued on card points. Observations and descriptions were made with an Olympus SZ61 binocular microscope under fluorescent lamps. Measurements were made with a binocular microscope (Olympus SZ40), and photographs were taken with a Sony 5000 digital camera attached to an Nikon SMZ 800N binocular microscope connected to a PC at IEBR. The scale-lines of the plates indicate in mm. Sculpture terms are based on Harris (1979), terminology used in this paper follows the modified Comstock-Needham system (van Achterberg 1993). For the identification of the East Palaearctic genera of Doryctinae see Belokobylskij and Maetô (2009); for division of *Rhaconotus* species groups see Belokobylskij (2001). Abbreviations used in this paper are as follows:

**OD** diameter of posterior ocellus;

**OOL** ocular-ocellar line;

**POL** postocellar line;

“**Doryc.+number**” code number indexing for specimens of the Doryctinae in the collection;

**MT** Malaise trap.

The holotypes are kept in the parasitoid collections of Department of Insect Ecology, the Institute of Ecology and Biological Resources, Ha Noi, Vietnam (**IEBR**).

## Systematics

### Checklist and distribution of *Rhaconotusjacobsoni* group species

*Rhaconotusceylonicus* Belokobylskij, 2001 / Sri Lanka

*Rhaconotusdirectus* Long, sp. nov. / Vietnam

*Rhaconotusjacobsoni* (Szepligeti, 1908) / Indonesia

*Rhaconotuslaevigatus* Long, sp. nov. / Vietnam

*Rhaconotuslongithorax* Belokobylskij, 2001 / Philippines

*Rhaconotusrobustus* Long, sp. nov. / Vietnam

*Rhaconotussimulatus* Long, sp. nov. / Vietnam

*Rhaconotusthayi* Belokobylskij, 2001 / China, Vietnam

### Key to species of *Rhaconotusjacobsoni* group from Vietnam

The Vietnamese species of *Rhaconotusjacobsoni* group are distinguished from other species by having the distance from pronotal carina to mesonotum equal or subequal to distance from carina to anterior margin of pronotum and can be inserted in the key by Belokobylskij (2001) as follows:

**Table:** 

1	Hind tibia entirely dark brown to black (female), yellow or brownish yellow (male); distance from carina to mesonotum equal to distance from carina to anterior margin of pronotum; vertex and mesonotum more or less with dense and long setae	**2**
–	Hind tibia entirely reddish yellow or yellow basally (female); distance from carina to mesonotum about 1.5 × distance from carina to anterior margin of pronotum; vertex and mesonotum with sparse and shorter setae	**three species of *R.jacobsoni* group** ^1^
2	Metanotum in lateral view with long pointed tooth (Fig. 5a); vein cu-a almost interstitial, vein 1-CU1 nearly quadrate (Fig. 10); propodeum with short median carina in basal 0.3; basolateral area of propodeum not emarginate posteriorly (Fig. 12); second tergite without lenticular apical area (Fig. 8). Body length 6.5 mm	***R.directus* Long, sp. nov.**
–	Metanotum in lateral view with short pointed tooth; vein cu-a distinctly postfurcal; vein 1-CU1 equal or subequal to vein cu-a (Figs 23, 35, 46); propodeum with median carina in basal 0.5–0.6 (Figs 21, 32, 44); basolateral area of propodeum emarginate posteriorly (not emarginated in *R.thayi* and *robustus*); second tergite with lenticular apical area (Figs 19, 43)	**3**
3	Male, hind tibia yellow or brownish yellow (Fig. 34); vertex and mesonotum with sparse short setae (Figs 26, 30); hind femur robust, 2.75 × its maximum width (Fig. 34); propodeum without posterior emarginate areola, almost foveolate-rugose apically (Fig. 32). Body length 6.2 mm	***R.robustus* Long, sp. nov.**
–	Female, hind tibia entirely black or blackish brown (Fig. 49); vertex and mesonotum with rather dense and long setae (Figs 14, 16, 40, 42); hind femur slender, 3.2–4.5 × its maximum width (Figs 20, 48); propodeum with posterior emarginate areola (Figs 21, 44)	**4**
4	Second submarginal cell of fore wing long, basal length 4.2 × its maximum width (Fig. 23); hind femur rather long, length 4.5 × as long as its maximum with (Fig. 20); mesopleuron almost smooth (Fig. 18); first metasomal tergite almost granulate coriaceous, sparsely striate apically (Fig. 19). Body length 7.5 mm	***R.laevigatus* Long, sp. nov.**
–	Second submarginal cell of fore wing shorter, basal length 3.2–3.5 × its maximum width (fig. 46, fig. 131 in Belokobylskij, 2001); hind femur slender, length 3.2–3.4 × its maximum with (fig. 48, fig. 134 in Belokobylskij, 2001); mesopleuron granulate or granulate coriaceous; first metasomal tergite coarsely striate, granulate between striae (fig. 43, fig. 135 in Belokobylskij, 2001)	**5**
5	Mesosoma 2.7–2.9 × as long as high; precoxal sulcus wide, crenulate (fig. 129 in Belokobylskij, 2001); basolateral area of propodeum not emarginate posteriorly; length of first metasomal 2.5–2.8 × apical width (fig. 135 in Belokobylskij, 2001). Body length 5.7–8.1 mm; frons coarsely rugose. Body length 5.7–8.1 mm	***R.thayi* Belokobylskij**
–	Mesosoma 2.5 × as long as high; precoxal sulcus narrow, punctate (Fig. 42); basolateral area of propodeum emarginate posteriorly, foveolate-rugose apically (Fig. 44); length of first metasomal 2.2 × apical width (Fig. 43); frons finely granulate. Body length 6.7 mm	***R.simulatus* Long, sp. nov.**

### Descriptions of species

#### Rhaconotus directus

Taxon classificationAnimaliaHymenopteraBraconidae

Long sp. nov.

http://zoobank.org/58A6D8BA-7804-43BE-93A3-0B80975B4D18

Figs 1 , 2–12

##### Material.

Holotype, female, “Doryc.035”, (IEBR), NW Vietnam: Hoa Binh, Mai Chau, Pa Co, forest, 1100 m, 26.iv.2002, KD Long.

##### Diagnosis.

Occipital carina complete medio-dorsally, obliterated below and not fused with hypostomal carina (Fig. 3); frons almost flat, finely granulate; vertex and temple finely granulate; distance from pronotal carina to mesonotum equal to distance from carina to anterior margin of pronotum; in lateral view, metanotum with long pointed tooth (Fig. 5a); precoxal sulcus long, narrow, crenulate (Fig. 5); mesopleuron and metapleuron finely granulate; notauli shallow, sparsely crenulate anteriorly, widened posteriorly, with median crenulate depression (Fig. 6); propodeum with median carina in basal 0.3; pterostigma 4.5 × as long as wide; fore wing vein 3-SR 5.0 × vein r; vein 1-CU1 very short, nearly quadrate; vein cu-a almost interstitial (Fig. 10); second submarginal cell parallel-sided, basal length 2.9 × its maximum width and 0.9 × subdiscal cell (Fig. 10); vein 1-M of hind wing 6.3 × vein 1r-m (Fig. 11); inner side of fore tibia with four spines; hind coxa finely and densely granulate; hind tibia 7.7 × its maximum width; first metasomal tergite gradually widened from base to apex, 2.4 × its apical width (Fig. 8); second tergite with lenticular apical area weakly delineated with shallow furrows (Fig. 8); second-third tergites coarsely longitudinally striate; fourth-fifth tergites longitudinally striate basally, finely striate apically; sixth tergite with semi-circular striae in its apical half.

##### Description.

**Figure 1.**
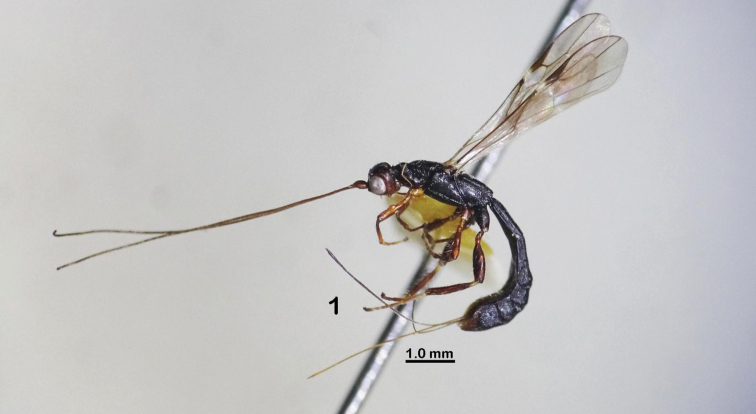
*Rhaconotusdirectus* Long, sp. nov., female, holotype (habitus, lateral view).

Female, body length 5.4 mm; fore wing length 4.5 mm; ovipositor sheath 3.4 mm (Fig. 1).

**Figures 2–12.**
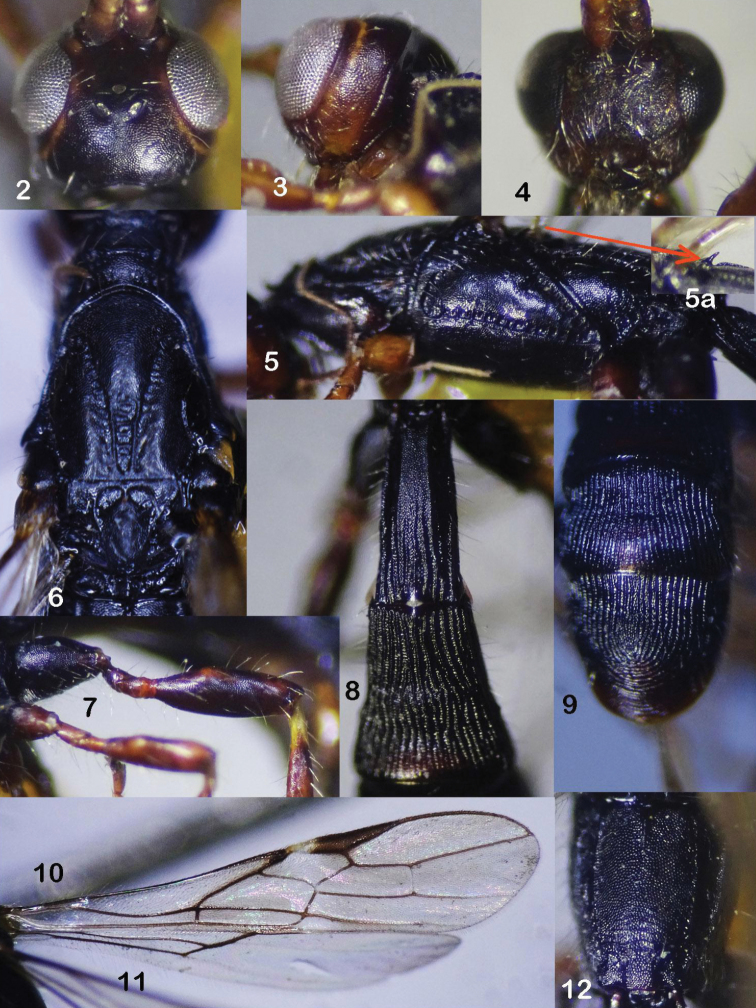
*Rhaconotusdirectus* Long, sp. nov., female, holotype **2** head, dorsal view **3** head, lateral view **4** head, frontal view **5** mesopleuron **6** mesonotum, dorsal view **7** hind coxa and femur **8** metasomal tergites 1–3, dorsal view **9** metasomal tergites 5–6 **10** fore wing **11** hind wing **12** propodeum.

***Head.*** Antenna incomplete, with 45 segments remaining; scapus length dorsally 1.8 × as long as its maximum width; third antennal segments 1.1 × as long fourth segment; in dorsal view, temple roundly narrowed behind eye; head width 1.3 × its median length; median length of head 3.0 × as long as temple; height of eye 1.9 × temple (Fig. 2); in lateral view, transverse diameter of eye 1.4 × length of temple (13 : 9); eye length 1.4 × its width (18 : 13) (Fig. 3); ocelli small, basal side of ocellar triangle 1.5 × lateral sides; POL 1.5 × OD and 0.5 × OOL (Fig. 2); in frontal view, eye 2.1 × as high as broad; malar space height 0.5 × height of eye, and 1.3 × basal width of mandible; face width 1.3 × height of eye, and 1.8 × height of face and clypeus combined (Fig. 4); hypoclypeal depression width 1.3 × as long distance from edge of depression to eye, 4.0 × as wide as face, and 1.6 × as wide as basal width of mandible; distance between tentorial pits 1.5 × as long as long distance from pit to eye; occipital carina complete medio-dorsally, obliterated below and not fused with hypostomal carina above base of mandible (Fig. 3); length of maxillary palp 1.45 × height of head (without mandible); frons almost flat, finely granulate; vertex and temple finely granulate; face setose, granulate (Fig. 4).

***Mesosoma.*** Distance from pronotal carina to mesonotum equal to distance from carina to anterior margin of pronotum; length of mesosoma 2.9 × its height (Fig. 5); in lateral view, metanotum with long pointed tooth (Fig. 5a); pronotal trough crenulate anteriorly, with transverse striae posteriorly; precoxal sulcus long, narrow, crenulate (Fig. 5); mesopleuron and metapleuron finely granulate; mesoscutum finely granulate; notauli shallow, sparsely crenulate anteriorly, widened posteriorly with crenulate depression (Fig. 6); scutellar depression 0.4 × as long as scutellum; scutellum finely granulate (Fig. 6); propodeum with lateral carinae, median carina in basal 0.3 of propodeum (Fig. 12); propodeum almost finely granulate; apex of propodeum with 2–3 transverse rugosities (Fig. 12).

***Wings.*** Fore wing 4.35 × as long as its maximum width; pterostigma 4.5 × as long as wide; vein r arising from middle of pterostigma; vein 1-R1 1.2 × as long as pterostigma; vein 3-SR 5.0 × vein r, and 0.5 × vein SR1, and 1.4 × vein 2-SR; vein m-cu postfurcal; second submarginal cell of fore wing parallel-sided, basal length 2.9 × as long as its maximum width (Fig. 10), and 0.9 × as long as subdiscal cell; subdiscal cell roundly closed on level of vein m-cu; vein 1-CU1 very short, nearly quadrate; vein cu-a almost interstitial (Fig. 10); hind wing 5.0 × as long as its maximum width; vein M+CU 0.3 × vein 1-M ; vein 1-M 6.3 × vein 1r-m (Fig. 11).

***Legs.*** Fore tarsus 1.2 × as long as fore tibia; inner side of fore tibia with four spines; hind coxa with baso-ventral tooth (Fig. 7), finely and densely granulate; hind femur, tibia and basitarsus 3.3, 7.7, and 6.7 × their maximum width, respectively; dorsal side of hind femur with short sparse semi-erected setae (Fig. 7), length of seta about 0.5 × as long as maximum width of femur; outer side of hind tibia with sparse semi-erected setae, length of seta as long as maximum width of hind tibia; inner hind tibial spur 0.2 × as long as hind basitarsus; hind tarsus 0.9 × as long as hind tibia; basitarsus 0.7 × as long as second-fifth tarsal segments combined (20:33); second tarsal segment 0.5 × as long as basitarsus (10 : 20), and 2.0 × as long as fifth tarsal segment (without pretarsus); fourth tarsal segment 0.6 × fifth tarsal segment.

***Metasoma.*** Metasoma 1.4 × as long as head and mesosoma combined; first tergite gradually widened from base to apex; maximum width of first tergite 1.4 × its minimum width (Fig. 8); length of first metasomal tergite 2.4 × apical width, and 1.45 × length of propodeum; second suture indistinct because of straight longitudinal striae; second tergite with lenticular apical area weakly delineated with wide shallow furrows (Fig. 8); second tergite with apical area 2.45 × as long as length of third tergite (Fig. 8); first metasomal tergite with dorsal carinae, granulate basally, longitudinally striate apically (Fig. 8); second-third tergites coarsely longitudinally striate; fourth-fifth tergites largely longitudinally striate basally, finely striate apically; sixth tergite with semi-circular striae in its apical half (Fig. 9).

***Colour.*** Body black; head dark brown; antenna brownish yellow, palpi brown, except apical segment of maxillary palp pale yellow; fore and middle legs brownish yellow, except tarsus yellow, hind coxa dark brown; hind femur and tibia brown; hind tarsus yellow; tegula brown; wing veins yellowish brown; pterostigma brown, cream white basally (Fig. 10).

##### Male.

Unknown.

##### Biology.

Unknown.

##### Etymology.

From *directus* (Latin for “set straight”, “arrange in a straight line”), because of the interstitial vein cu-a of fore wing.

#### Rhaconotus laevigatus

Taxon classificationAnimaliaHymenopteraBraconidae

Long sp. nov.

http://zoobank.org/FD83916A-D100-4251-95BE-9D1990976C66

Figs 13 , 14–24

##### Material.

Holotype, female, “Doryc.080” (IEBR), NE Vietnam: Vinh Phuc, Me Linh, Tam Dao foothill, forest, 13.v.2002, KD Long.

##### Diagnosis.

Occipital carina finely complete medio-dorsally, not fused with hypostomal carina above base of mandible (Fig. 16); frons almost flat, with transverse fine striae (Fig. 14); vertex and temple shiny, smooth; distance from pronotal carina to mesonotum equal to distance from carina to anterior margin of pronotum; in lateral view, metanotum with short pointed tooth; precoxal sulcus narrow, almost smooth (Fig. 18); mesopleuron and metapleuron finely granulate; notauli shallow, sparsely crenulate anteriorly, widened posteriorly, with median more or less shallow depression (Fig. 17); propodeum with median carina in basal 0.6 (Fig. 21); pterostigma 3.3 × as long as wide; fore wing vein 3-SR 3.0 × vein r; vein 1-CU1 0.05 × vein 2-CU1; basal length of second submarginal cell 2.9 × its maximum width and 0.9 × subdiscal cell (Fig. 23); vein 1-M of hind wing 4.2 × vein 1r-m (Fig. 24); inner side of fore tibia with six spines; hind coxa finely and densely granulate; hind tibia 11.1 × its maximum width; first metasomal tergite 2.7 × its apical width, granulo-coriaceous; second tergite with lenticular apical area delineated with furrows (Fig. 19); second-third tergites coarsely longitudinally striate, but finely striate apically; fourth tergite largely striate basally contrast to fine striate apex; fifth tergite striate medially, granulo-punctate apically; sixth tergite fine basally, finely rugose medially, with fine semi-circular striae at apex (Fig. 22).

##### Description.

**Figure 13.**
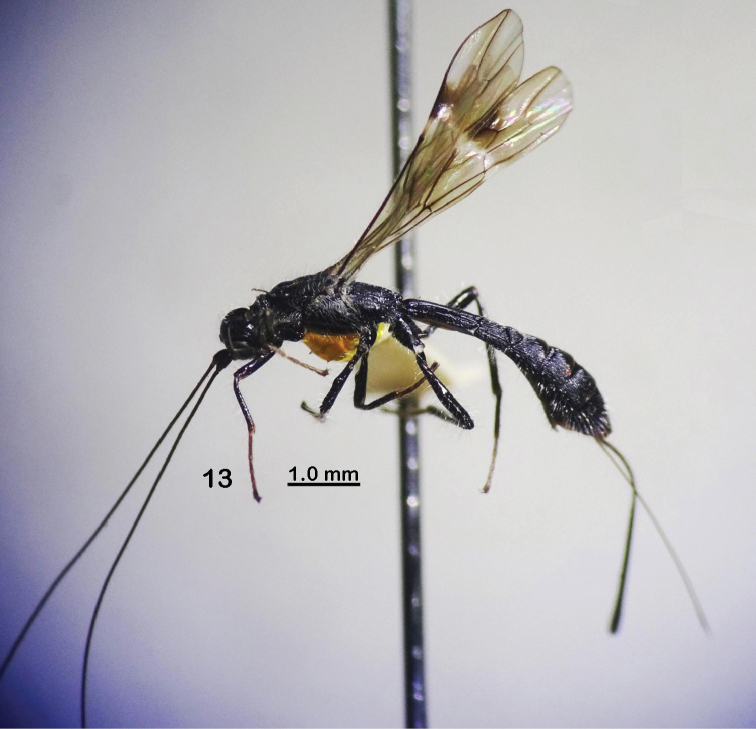
*Rhaconotuslaevigatus* Long, sp. nov., female, holotype (habitus, lateral view).

Female, body length 7.6 mm; fore wing length 5.7 mm; ovipositor sheath 3.9 mm (Fig. 13).

**Figures 14–24.**
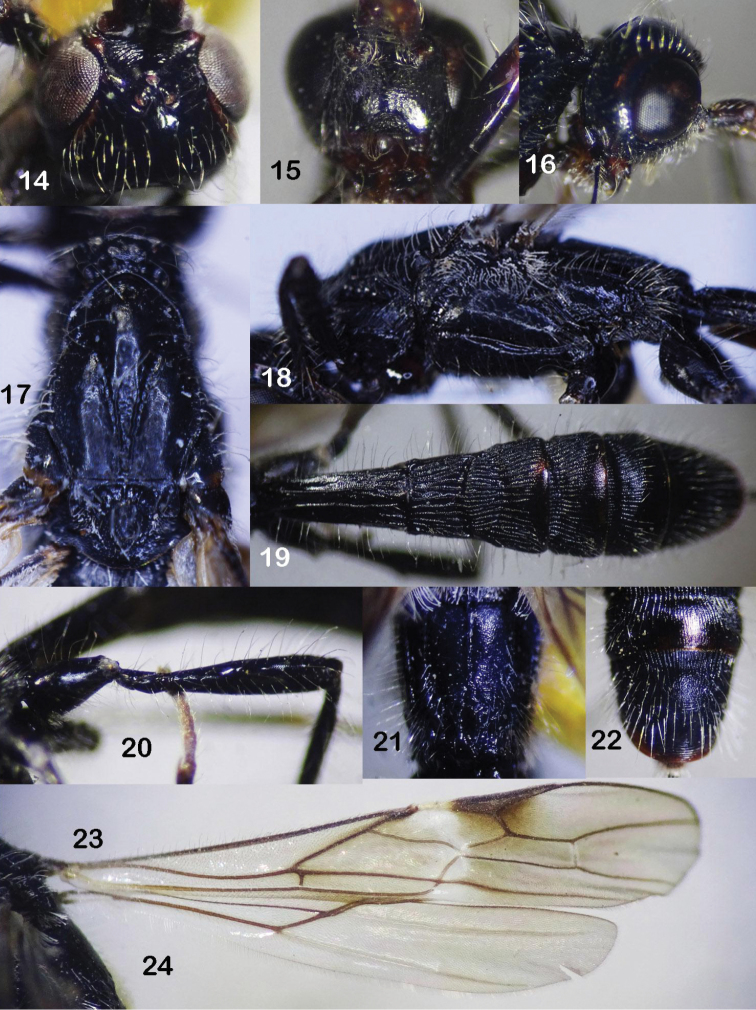
*Rhaconotuslaevigatus* Long, sp. nov., female, holotype **14** head, dorsal view **15** head, frontal view **16** head, lateral view **17** mesonotum, dorsal view **18** mesopleuron **19** metasomal tergites 1–4, dorsal view **20** hind coxa and femur **21** propodeum **22** metasomal tergites 5–6, dorsal view **23** fore wing **24** hind wing.

*Head*. Antenna incomplete, with 54 segments remaining; scapus length dorsally 1.5 × as long as its maximum width; third antennal segment 1.1 × as long fourth segment; in dorsal view, temple roundly narrowed behind eye; median length of head 2.7 × as long as temple; height of eye 1.6 × as long as temple (Fig. 14); in lateral view, transverse diameter of eye 1.5 × length of temple; eye 1.2 × longer than its width (Fig. 16); ocelli small, basal side of ocellar triangle 1.5 × lateral sides; POL 1.5 × OD, and 0.4 × OOL (Fig. 14); in frontal view, eye twice as high as broad; malar space 0.5 × height of eye, and 1.25 × as long as basal width of mandible; face width 1.1 × height of eye, and 1.4 × height of face and clypeus combined (Fig. 15); hypoclypeal depression as long as distance from edge of depression to eye, 0.5 × as wide as face, and 1.5 × as wide as basal width of mandible; distance between tentorial pits 1.6 × as long as long distance from pit to eye; occipital carina finely complete medio-dorsally, not fused below with hypostomal carina above base of mandible (Fig. 16); head below eyes roundly narrowed below eyes (Fig. 14); length of maxillary palp 1.4 × height of head (without mandible); frons almost flat, with transverse fine striae anteriorly, smooth posteriorly; vertex and temple shiny smooth; face setose, finely punctate (Fig. 15).

*Mesosoma*. Distance from pronotal carina to mesonotum equal to distance from carina to anterior margin of pronotum; mesoscutum more or less depressed posteriorly; length of mesosoma 2.6 × its height (Fig. 18); in lateral view, metanotum with short pointed tooth; pronotal side deep, almost smooth; mesoscutum granulo-coriaceous; notauli narrow, punctate (Fig. 17); scutellar depression 0.4 × as long as scutellum, with one median carina (Fig. 17); scutellum finely granulate; precoxal sulcus narrow, smooth (Fig. 18); mesopleuron almost smooth; subalar depression wide, deep, with sparse crenulae (Fig. 18); propodeum with carina in its basal 0.6 (Fig. 21).

*Wings*. Fore wing 4.7 × as long as its maximum width; pterostigma 3.3 × as long as wide; vein r arising behind middle of pterostigma (distance from apex of pterostigma to vein r 0.8 × distance from vein r to base of pterostigma); vein 1-R1 1.1 × as long as pterostigma (Fig. 23); vein 3-SR 3.0 × vein r, 0.2 × vein SR1, and 1.5 × vein 2-SR; vein m-cu distinctly postfurcal; basal length of second submarginal cell of fore wing 4.7 × as long as its maximum width (42 : 9), and 0.9 × as long as subdiscal cell; subdiscal cell roundly closed on level of vein m-cu; vein 1-CU1 0.7 × vein cu-a, and 0.05 × vein 2-CU1 (Fig. 23); hind wing 6.1 × as long as wide; vein M+CU 0.2 × vein 1-M; vein 1-M 4.2 × vein 1r-m (Fig. 24).

*Legs*. Fore tarsus 1.4 × as long as fore tibia; inner side of fore tibia with six spines; outside of fore tibia with long erected setae, length of seta twice as long width of fore tibia; hind coxa with baso-ventral tooth; hind femur, tibia and basitarsus 5.6, 11.1 and 8.0 × their maximum width, respectively; dorsal side of hind femur with long semi-erected setae, length of seta 1.6 × as long as maximum width of hind tibia (Fig. 20); outside of hind tibia with long erected setae, length of seta twice as long as maximum width of hind tibia; inner hind tibial spur 0.3 × as long as hind basitarsus; hind tarsus 1.2 × as long as hind tibia; basitarsus 0.8 × as long as second-fifth tarsal segments combined; second tarsus 0.4 × as long as basitarsus, and 1.4 × as long as fifth tarsus (without pretarsus); fourth tarsus 0.6 × fifth tarsus; hind coxa with sparse setae, finely granulate.

*Metasoma*. Metasoma 1.5 × as long as head and mesosoma combined; maximum width of first tergite 1.5 × its minimum width; length of first metasomal tergite 2.7 × apical width, and 1.7 × length of propodeum; second tergite with lenticular apical area separated with distinct wide crenulate furrow (Fig. 19); length of separated area 0.75 × length of second tergite, and 0.9 × third tergite; length of second tergite 0.6 × as long as its basal width, and 1.2 × length of third tergite; first metasomal tergite with long straightly erected setae laterally, with two almost parallel dorsal carina running from base to apex (Fig. 19); first tergite almost granulo-coriaceous; second tergite coarsely striate; third-fourth tergites largely striate basally, finely striate apically (Fig. 19); fifth tergite striate basally, granulo-punctate apically; sixth tergite setose, finely striate basally, finely rugose medially, with fine semi-circular striae apically (Fig. 22);

*Colour*. Black, antenna brown; palpi brown; all legs dark brown to black, expect tarsus yellowish brown; tegula brown; wing veins brown; pterostigma brown, cream white basally, surrounding vein r beneath pterostigma smoky brown (Fig. 23); ovipositor sheath brown.

##### Male.

Unknown.

##### Biology.

Unknown.

##### Etymology.

From *laevis* (Latin for smooth, polished), because of vertex, temple and mesopleuron shiny smooth.

#### Rhaconotus robustus

Taxon classificationAnimaliaHymenopteraBraconidae

Long sp. nov.

http://zoobank.org/368DE76C-6AA0-4FFC-A4DF-57384123A56F

Figs 25 , 26–36

##### Material.

Holotype, male, “Doryc.722” (IEBR), NW Vietnam: Hoa Binh, Mai Chau, Tan Son, orchard, MT, 20°43'10.3"N 104°59'47.0"E, 950m, 1-5.v.2010, KD Long.

##### Diagnosis.

Antenna with 46 segments (male); occipital carina finely complete medio-dorsally, fading below distal to hypostomal carina above base of mandible (Fig. 27); frons slightly depressed, with sparse fine striae (Fig. 26); vertex and temple shiny, smooth; distance from pronotal carina to mesonotum equal to distance from carina to anterior margin of pronotum; in lateral view, metanotum with short pointed tooth; precoxal sulcus narrow, straight, crenulate (Fig. 30); mesopleuron and metapleuron finely granulate; notauli shallow, sparsely crenulate anteriorly, slightly widened posteriorly, with two longitudinal convergent carinae running close to scutellar sulcus (Fig. 29); propodeum with baso-lateral areas emarginated by carina (Fig. 32); median carina in basal 0.5; pterostigma 4.4 × as long as wide; fore wing vein 3-SR 2.7 × vein r; vein 1-CU1 0.12 × vein 2-CU1 (Fig. 35); second submarginal cell slightly widened apically, basal length 3.1 × its maximum width and 1.6 × subdiscal cell; vein 1-M of hind wing 4.25 × vein 1r-m (Fig. 36); inner side of fore tibia with six spines; hind coxa finely granulate; hind tibia robust, 8.5 × its maximum width; first metasomal tergite nearly parallel-sided, 2.5 × its apical width (Fig. 33), finely granulate basally, longitudinally striate apically; second-fifth tergites longitudinally striate; sixth tergite rugo-striate basally, almost smooth apically (Fig. 31).

##### Description.

**Figure 25.**
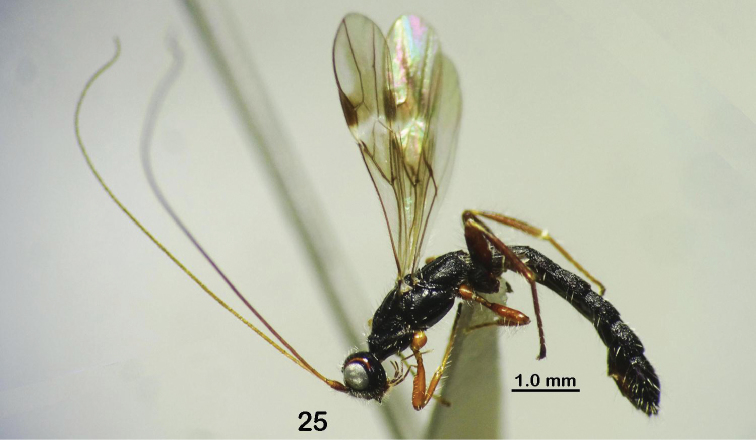
*Rhaconotusrobustus* Long, sp. nov., male, holotype (habitus, lateral view).

Male, body length 5.9 mm; fore wing length 3.7 mm (Fig. 25).

**Figures 26–36.**
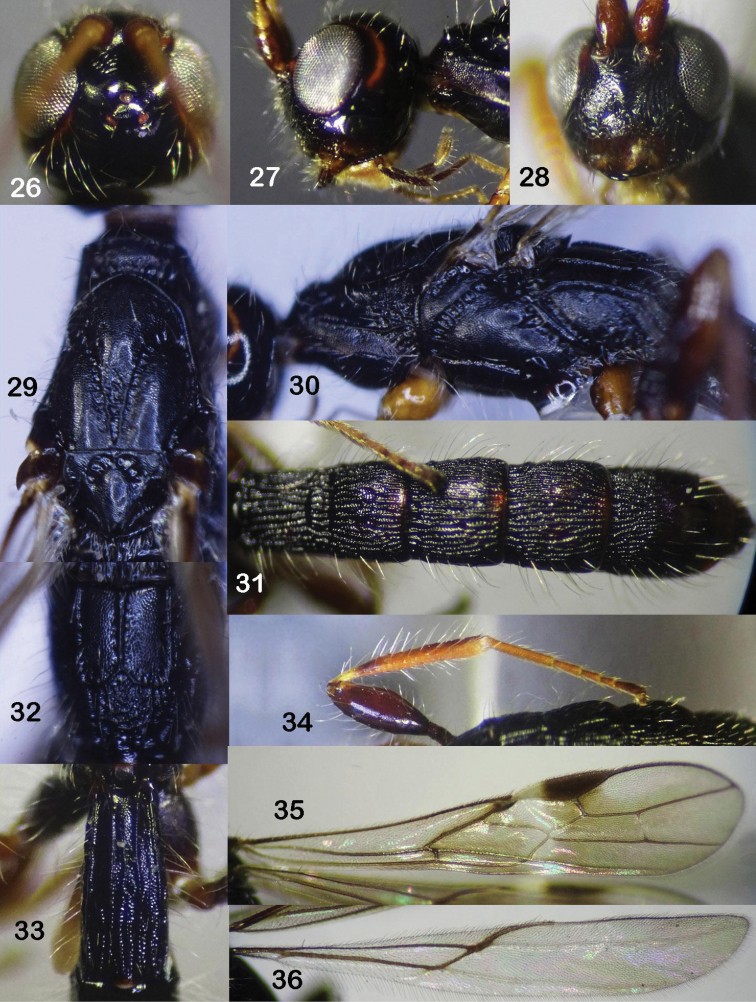
*Rhaconotusrobustus* Long, sp. nov., male, holotype **26** head, dorsal view **27** head, lateral view **28** head, frontal view **29** mesonotum, dorsal view **30** mesopleuron **31** tergites 2–6, dorsal view **32** propodeum **33** first metasomal tergite **34** hind femur and tibia **35** fore wing **36** hind wing.

*Head*. Antenna with 46 segments; scapus dorsally 1.3 × longer than its maximum width; third antennal segment 1.1 × as long fourth segment; in dorsal view; temple roundly behind eye; head width 1.3 × its median length (Fig. 26); median length of head 1.8 × as long as temple; height of eye 1.3 × temple; in lateral view, transverse diameter of eye 1.2 × length of temple; eye 1.4 × longer than its width (Fig. 27); ocelli small, basal side of ocellar triangle 1.5 × lateral sides; POL 1.5 × as long as OD, and 0.6 × OOL (Fig. 26); in frontal view, eye 2.3 × as high as broad; malar space 0.4 × height of eye, 1.3 × as long as basal width of mandible (Fig. 28); face width 1.1 × height of eye, and 1.3 × height of face and clypeus combined; width of hypoclypeal depression 0.8 × as long distance from edge of depression to eye, 0.4 × as wide as face, and 1.2 × as wide as basal width of mandible; distance between tentorial pits 1.1 × as long as long distance from pit to eye; occipital carina complete, fading below distal to hypostomal carina above base of mandible (Fig. 27); head with long sparse setae, roundly narrowed below eyes (Fig. 26); length of maxillary palp 1.4 × height of head (without mandible); frons slightly depressed medially, with sparse fine striae; vertex and shiny, smooth (Fig. 26); face rugo-coriaceous; clypeus rugose (Fig. 28).

*Mesosoma*. Mesosoma depressed, its dorsal side almost flat; length 2.1 × its height (Fig. 30); pronotum with median transverse carina; notauli narrow, sparsely crenulate anteriorly, slightly widened posteriorly with two longitudinal convergent carinae running close to scutellar sulcus (Fig. 29); pronotal side almost flat, coriaceous medially, coarsely rugose posteriorly, finely granulate ventrally; precoxal sulcus long, narrow, crenulate; subalar depression largely crenulate; mesopleuron finely granulate (Fig. 30); mesoscutum and scutellum finely granulate; propodeum with baso-lateral areas emarginated by carinae (Fig. 32); median carina in basal 0.5 of propodeum; baso-lateral area finely granulate; propodeal areola rugose.

*Wings*. Fore wing 4.1 × as long as its maximum width; pterostigma 4.4 × as long as wide; vein r arising behind middle of pterostigma (distance from apex of pterostigma to vein r 0.7 × distance from vein r to base of pterostigma); vein 1-R1 1.2 × as long as pterostigma; vein 3-SR 2.7 × vein r, and 0.6 × vein SR1, and 1.3 × vein 2-SR; vein m-cu distinctly postfurcal; second submarginal cell of fore wing slightly widened apically (Fig. 35), basal length 3.1 × as long as its maximum width, and 1.6 × as long as subdiscal cell; subdiscal cell roundly closed on level of vein m-cu; vein 1-CU1 equal to vein cu-a, and 0.12 × vein 2-CU1 (Fig. 35); hind wing 6.7 × as long as wide; vein M+CU 0.3 × vein 1-M; vein 1-M 4.25 × vein 1r-m (Fig. 36).

*Legs*. Fore tarsus 1.5 × as long as fore tibia; inner side of fore tibia with six spines; hind coxa with baso-ventral tooth, finely granulate; hind femur robust (Fig. 34), length of hind femur, tibia and basitarsus 2.5, 8.5 and 10.5 × their maximum width, respectively; outer side of hind tibia with long semi-erected setae, length of seta 1.5 × maximum width of tibia (Fig. 34); inner hind tibial spur 0.3 × as long as hind basitarsus; hind tarsus 0.9 × as long as hind tibia; basitarsus 0.8 × as long as second-fifth tarsal segments combined; second tarsus 0.4 × as long as basitarsus, and 1.1 × as long as fifth tarsus (without pretarsus); fourth tarsus 0.6 × fifth tarsus.

*Metasoma*. Metasoma 1.3 × as long as head and mesosoma combined; first metasomal tergite nearly parallel-sided (Fig. 33); length of first metasomal tergite 2.5 × apical width, and 1.4 × length of propodeum; second metasomal tergite with narrow lenticular apical area separated with shallow crenulate furrow, length of lenticular apical area 0.3 × length of second tergite (Fig. 31); length of second tergite 0.9 × as long as its basal width, and 0.7 × length of third tergite; first metasomal tergite with two dorsal carinae in basal 0.5 of tergite; finely granulate basally, longitudinally striate apically (Fig. 33); second-fifth tergites longitudinally striate; sixth tergite sparsely setose, rugo-striate basally, almost smooth apically (Fig. 31).

*Colour*. Body black, antenna yellow, but brown apically; scapus brownish yellow; head black with yellow semi-circular stripe around eye dorso-laterally (Fig. 27); palpi brown, except apical segment of labial palp and two apical segments of maxillary palp yellow; fore and middle legs yellow; hind coxa and femur yellowish brown; hind tibia and tarsus yellow; tegula brownish yellow; wing veins pale brown; pterostigma pale brown, cream white basally (Fig. 35); wing membrane hyaline with smoky spots medially.

##### Female.

Unknown.

##### Biology.

Unknown.

##### Etymology.

From *robustus* (Latin for strong), because of the robust hind femur.

#### Rhaconotus simulatus

Taxon classificationAnimaliaHymenopteraBraconidae

Long sp. nov.

http://zoobank.org/B62850AA-A9AA-4B94-B95D-C22AE73A9E90

Figs 37 , 38–49

##### Material.

Holotype, female, “Doryc.791” (IEBR), NW Vietnam: Son La, orchard, MT, 21°18'03.6"N 103°55'38.3"E, 671 m, 15–25.vi.2016, KD Long.

##### Diagnosis.

Occipital carina finely complete medio-dorsally, fused with hypostomal carina above base of mandible (Fig. 40); frons slightly depressed medially, rugose anteriorly, finely granulate posteriorly; vertex and temple smooth; distance from pronotal carina to mesonotum equal to distance from carina to anterior margin of pronotum; in lateral view, metanotum with short pointed tooth; precoxal sulcus long, sinuate, crenulate (Fig. 42); mesopleuron finely granulate; notauli narrow, widened anteriorly, crenulate with two posterior convergent carinae running close to scutellar sulcus (Fig. 41); propodeum with median carina in basal 0.5; pterostigma 5.7 × as long as wide; fore wing vein 3-SR 3.4 × vein r; vein 1-CU1 0.08 × vein 2-CU1 (Fig. 46); basal length of second submarginal cell 3.8 × its maximum width and as long as subdiscal cell; vein 1-M of hind wing 4.4 × vein 1r-m (Fig. 47); inner side of fore tibia with five spines; hind coxa finely granulate; hind tibia 8.3 × its maximum width; first metasomal tergite 2.2 × its apical width (Fig. 43), longitudinally striate in apical 0.7; furrow between second and third tergites wide, crenulate; third tergite largely striate basally, densely and finely striate apically; fourth-fifth tergites longitudinally striate; sixth tergite striate basally, with fine semi-circular striae at apex (Fig. 45).

##### Description.

**Figures 37.**
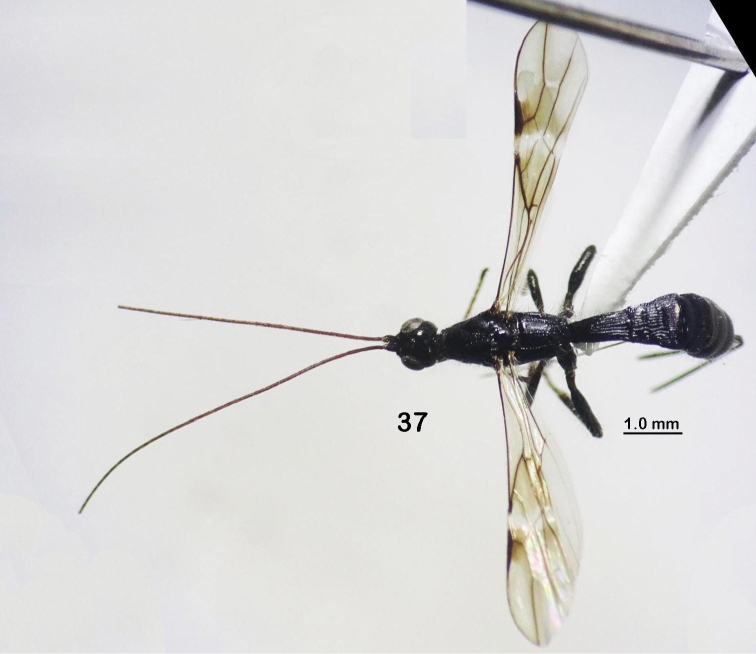
*Rhaconotussimulatus* Long, sp. nov., female, holotype (habitus, dorsal view).

Female, body length 6.6 mm; fore wing length 5.0 mm; ovipositor sheath 2.4 mm (Fig. 37).

**Figures 38–49.**
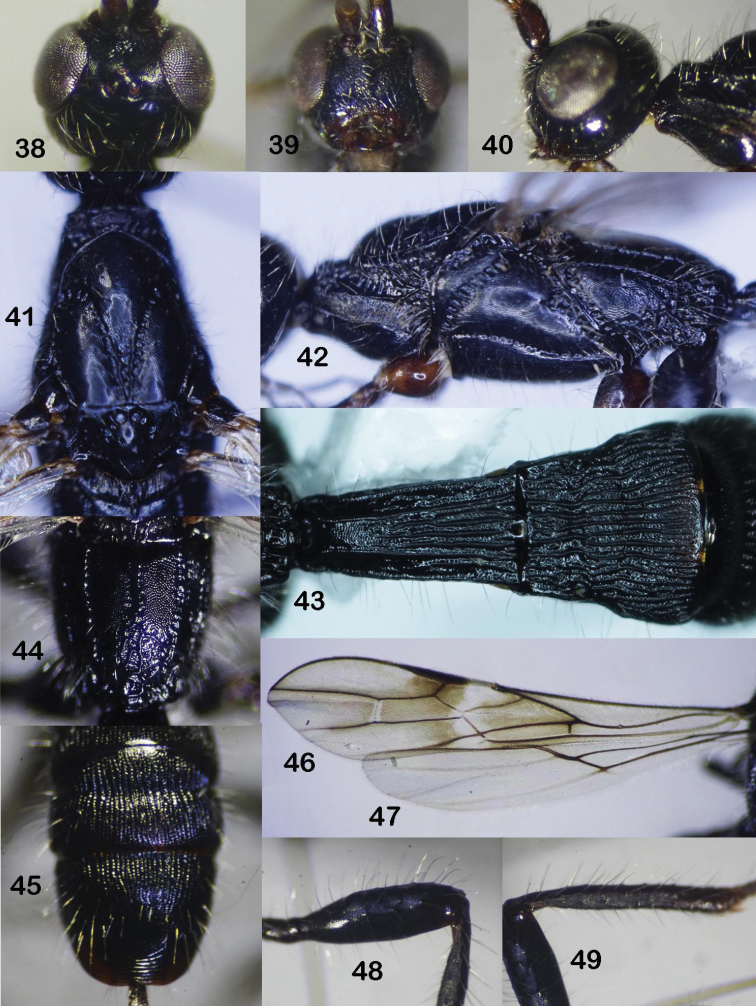
*Rhaconotussimulatus* Long, sp. nov., female, holotype **38** head, dorsal view **39** head, frontal view **40** head, lateral view **41** mesonotum, dorsal view **42** mesopleuron **43** metasomal tergites 1–3, dorsal view **44** propodeum **45** metasomal tergites 5–6, dorsal view **46** fore wing **47** hind wing **48** hind femur **49** hind tibia.

*Head*. Antenna incomplete, with 30 segments remaining; scapus length dorsally 1.7 × its maximum width; third antennal segment 1.3 × as long fourth segment; in dorsal view, temple roundly narrowed behind eye; head width 1.3 × its median length (Fig. 38); median length of head 3.2 × as long as temple; height of eye 1.09 × temple; in lateral view, eye 1.3 × longer than width; transverse diameter of eye 1.6 × length of temple (Fig. 40); ocelli small, basal side of ocellar triangle 1.5 × lateral sides; POL as long as OD, and 0.5 times OOL; in frontal view, eye 2.1 × as high as broad (Fig. 39); malar space height 0.5 × height of eye, and as long as basal width of mandible; face width 1.2 × height of eye, and 1.3 × height of face and clypeus combined (Fig. 39); hypoclypeal depression width 0.6 × as long distance from edge of depression to eye, 0.4 × as wide as face; and 1.2 × as wide as basal width of mandible; distance between tentorial pits as long as long distance from pit to eye; occipital carina complete medio-dorsally, fused bellow with hypostomal carina distal to base of mandible (Fig. 40); maxillary palp 1.3 × height of head (without mandible); frons slightly depressed medially, rugose anteriorly, granulo-coriaceous posteriorly; vertex between lateral ocellus and eye margin finely granulate, vertex between lateral ocellus and eye margin finely granulate; vertex below posterior ocelli, temple smooth; face latero-ventrally and malar space coriaceous; clypeus rugose.

*Mesosoma*. Length of mesosoma 2.4 × its height (Fig. 42); in lateral view, metanotum with short pointed tooth; notauli narrow, widened anteriorly, crenulate with two posterior convergent carinae running close to scutellar sulcus (Fig. 41); scutellar sulcus with three carinae, 0.4 × as long as scutellum (Fig. 41); scutellum mesoscutum and scutellum finely granulate; pronotal side depressed medially, sparsely crenulate medially and anteriorly, coarsely rugose posteriorly, granulate ventrally; precoxal sulcus long, sinuate, crenulate (Fig. 42); mesopleuron finely granulate; subalar depression largely crenulate; propodeum with baso-lateral areas emarginated by carinae (Fig. 44); median carina in basal 0.5 of propodeum; baso-lateral areas finely granulate; propodeum coarsely rugose apically.

*Wings*. Fore wing 4.2 × as long as its maximum width; pterostigma 5.7 × as long as wide; vein r arising from middle of pterostigma; vein 1-R1 1.4 × as long as pterostigma; vein 3-SR 3.4 × vein r, 0.45 × vein SR1, and 2.0 × vein 2-SR; vein m-cu distinctly postfurcal; second submarginal cell parallel-sided, basal length 3.8 × as long as its maximum width, and as long as subdiscal cell (Fig. 46); subdiscal cell roundly closed on level of vein m-cu; vein 1-CU1 0.08 × vein 2-CU1, and 0.75 × vein cu-a; hind wing 7.0 × as long as wide; vein M+CU 0.3 × vein 1-M; vein 1-M 4.4 × vein 1r-m (Fig. 47).

*Legs*. Fore tarsus 1.6 × as long as fore tibia; inner side of fore tibia with five spines; hind coxa with baso-ventral tooth; hind coxa finely granulate; hind femur, tibia and basitarsus 3.0, 8.3 and 5.75 × their maximum width, respectively (Figs 48, 49); hind femur finely granulate; hind tibia with long semi-erected setae, length of seta as long as maximum width of tibia (Fig. 49); inner hind tibial spur 0.3 × as long as hind basitarsus; hind tarsus 0.8 × as long as hind tibia; basitarsus 0.7 × as long as second-fifth tarsal segments combined; second tarsus 0.4 × basitarsus, and as long as fifth tarsus (without pretarsus); fourth tarsus 0.2 × fifth tarsus.

*Metasoma*. Metasoma 1.25 × as long as head and mesosoma combined; first metasomal tergite distinctly widened at apex, with two dorsal carinae in whole length of tergite (Fig. 43); maximum width of first tergite 1.2 × its minimum width; length of first metasomal tergite 2.2 × apical width (Fig. 43), and 1.4 × length of propodeum; second tergite with more or less distinct lenticular apical area separated by furrows (Fig. 43); length of second tergite 0.44 × as long as its basal width, and 0.5 × length of third tergite; first metasomal tergite longitudinally striate in apical 0.7 of tergite (Fig. 43); furrow between second and third tergites wide, crenulate; third tergite largely striate basally, densely and finely striate apically; fourth-fifth tergites longitudinally striate (Fig. 43); sixth tergite striate basally, with fine semi-circular striae apically (Fig. 45);

*Colour*. Body black; antenna pale brown; palpi brown, except apical segment of maxillary palp pale yellow; fore coxa brownish yellow, fore femur and tibia yellowish brown; fore tarsus yellow; middle coxa yellowish brown; middle femur and tibia brown; middle tarsus yellow; hind leg brown, except tarsus brownish yellow; tegula brown; wing veins brown; pterostigma brown, cream white basally (Fig. 46); wing membrane yellow with brown clouds medially; ovipositor sheath brown.

##### Male.

Unknown.

##### Biology.

Unknown.

##### Etymology.

From *simulo* (Latin for imitate, copy), because this new species is similar to *R.thayi* Belokobylskij.

##### Remarks.

*R.simulatus*, sp. nov. is similar to *R.thayi* Belokobylskij, 2001, from China and Vietnam, but the new species differs from the later by having: a. Occipital carina fused bellow with hypostomal carina distal to base of mandible (Fig. 40; not fused in *R.thayi*); b. Vein 1-R1 of fore wing 1.4 × as long as pterostigma (1.1 × in *R.thayi*), and vein 3-SR 3.4 × vein r (4.0–4.8 × in *R.thayi*); c. First metasomal tergite with dorsal carinae in whole length of tergite (in basal third in *R.thayi*) and d. Precoxal sulcus sinuate, crenulate (straight and smooth medially in *R.thayi*).

## Supplementary Material

XML Treatment for Rhaconotus directus

XML Treatment for Rhaconotus laevigatus

XML Treatment for Rhaconotus robustus

XML Treatment for Rhaconotus simulatus

## Footnotes

see couplet 22 of the key by Belokobylskij (2001)
